# The Yangian relations of Heisenberg spin chain model

**DOI:** 10.1038/s41598-021-94050-6

**Published:** 2021-07-16

**Authors:** Guijiao Du, Kang Xue, Chengcheng Zhou

**Affiliations:** 1 School of Science, Changchun University of Science and Technology, Changchun, 130022 China; 2 School of Physics, Northeast Normal University, Changchun, 130024 China

**Keywords:** Mathematics and computing, Quantum physics

## Abstract

In this paper, we investigate the Yangian relations of Heisenberg spin chain systems. Firstly, we consider the closed XXZ spin chain model, through the Heisenberg spin XXZ model, we found the Hamiltonians for one kind system of three adjacent partial particles interaction systems. The model’s constitution rules of energy levels and energy states which expand from the few-particle system to multi-particle system have good regularity. In this system, we found Yangian’s law and illustrate it through graphs. Secondly, we further consider the closed XXZ spin chain’s generalization of other three neighboring particles interaction systems from few-particle system to multi-particle system. Finally, we also discussed the laws of the three adjacent particles system of some models, they are the XXZ model with twist boundary condition, the open XXZ spin chain model and the XXZ model containing the next neighbor. In addition, not only XXZ model, XXX model, XY model and Ising model, but the relevant laws of spin-1 systems of these models were also discussed, they have similar rules to the XXZ model. Through calculation and research, the eigensystems of these models all have good Yangian and constitution laws.

## Introduction

According to the realization of symmetries, it becomes possible to study physical models, efficiently calculate observables and analyze physical system’s properties. Symmetry is a core concept in modern physics. Hermann Weyl the German mathematician was the first to realize the importance of normative symmetry and apply the symmetries approach to physics. In 1950s, Yang and Robert Mills realized that normative symmetry could determine the form of theoretical Lagrangian, then *SU*(2) normative theory was constructed, then symmetry theory is widely used in particle physics, quantum field theory and so on. In the most extreme case, a theory has as many as many degrees of freedom as many independent symmetries. This is a rough definition of the integrable model. The properties of integrable systems can be identified by revealing mathematical structures that have been studied for decades^1–4^.

The Yangian symmetry is not only the realization of integrability, but also the extension of Lie algebra in Physics. In 1985, through Drinfeld’s fundamental paper^5^, Yangian was introduced for the first time. Drinfeld believes Yangian is a non-commutative Hopf algebra, The Yangian algebra corresponding to Lie algebra is a deformation of Hopf algebra class. It is closely related to the Yang-Baxter equation which is one of the most important concepts underlying integrable models. In 1985, Drinfel wanted to find an effective method of constructing solutions when he studied quantum Yang-Baxter equation, inspired by the rational solution of Yang-Baxter equation, Drinfeld introduced Yangian^5–9^. Yangian algebra also could be obtained by R-matrix^9^. In the 1990s, Reshetikhin and L.Takhtadzhjan found a description about defining quantum group^10^, which can define a relation-defining system by quantum R-matrix. That is called the algebraic Bethe ansatz^6,11,12^. Yangian represents a central concept within the framework of the physical integrable model and its mathematic foundation. Yangian algebra has many implementations in some areas and physical models, such as Lie algebras^10,13–17^, the scattering matrices and reflection matrices of the Yangian^18,19^, the RTT realization of Yangian with D-type Lie algebra^20^, the theory of classical W-algebras and affine vertex algebras^21–23^ , two-dimensional example (Gross-Neveu model)^24^, Hubbard model^25–27^ and integrable spin chains^28^.

**Figure 1:**
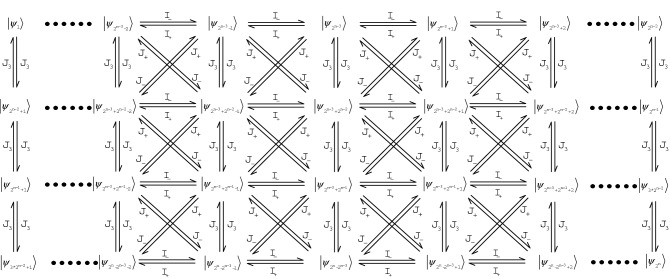
The Yangian and Lie algebra relations of three adjacent particle system in XXZ spin model with periodic boundary condition. This figure shows the transitions between states of the first, second and the last particles’ interaction of *N*-particle closed XXZ spin chain models. Lie algebra operators $I_{\alpha }$ represent transitions between states in the same energy level. Yangian operators $J_{\alpha }$ represent transitions between states in different energy states.

**Figure 2:**
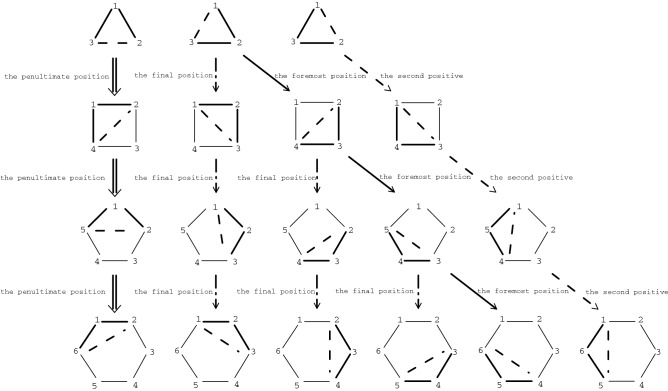
shows the generalization of the interaction model of any three adjacent particles of the closed XXZ spin chain model. In the action of three particles, the heavy solid line means that the action of two adjacent particles is additive, and the dotted line means that the action of two adjacent particles is subtractive. Figure 2 contains all the generalizations of closed XXZ spin chain.e The generalization rules: the double arrow indicates that the operator $\uparrow , \downarrow$ are added in the penultimate position to generalize, the dotted arrow indicates that the operators are added at the final position, the single arrow indicates that the operators are added at the foremost position, the dotted arrow indicates that the operators are added at the second positive.

During the research of spin chain’s Yangian symmetry, we considered four kinds of spin chain models: XXX spin chain, XXZ spin chain, XY spin chain and Ising model. We found Yangian rules in these systems when we considered three neighboring particles interactions in the system. In this paper, we study two kinds of boundary conditions, periodic boundary conditions and twist boundary conditions, the research systems are spin-$\frac{1}{2}$ and spin-1 system, during the study, we also consider the next nearest neighbor interaction of these models. This paper is organized as follows: in “Yangian of closed XXZ spin chain of N particles” section, we consider three particles interaction system in the closed XXZ spin chain model. Corresponding eigenvalues and eigenstate laws have also been found. The conditions of Yangian and Lie algebra of XXZ spin chain will be illustrated in Fig. 1 and corresponding formula representations will also be given. In “Yangian and eigensystem laws of closed XXZ spin chain of N particles” section, we found the way to construct the three particles interaction Hamiltonian by XXZ spin chain Hamiltonian, different construction methods correspond to different rules. The construction modes and laws of XXZ spin chain Hamiltonians are shown and illustrated in Fig. 2. The XXX spin chain, XY spin chain model and Ising model’s Yangian rules also be studied in “Studies on the laws of XXX model, XXZ model with other conditions and other models” section. Finally, we will give a summary in “Summary” section.

## Yangian of closed XXZ spin chain of N particles

In this section, we consider three particles interaction of closed XXZ spin chain model, the research contents are the rules of Lie algebra and Yangian of this model, eigensystem rules also be studied. The interaction between the first, second and the last particles are considered in this part, the interaction law of the other three particles will be shown in the next part. According to the study contents, we will recall the Lie algebra and Yangian. As we know, symmetry is an important concept in physics, since 1985, V. G. Drinfeld established Yangian theory, numerous studies have found that many physical models are Yangian symmetry. There are close relations between Lie algebra and Yangian, any simple Lie algebra *A* could associate with Hopf algebra *Y*(*A*) which is a deformation of the universal enveloping algebra for the polynomial current Lie algebra. In this paper, we use the Yangian operator $J^{\alpha },(\alpha =\pm ,3)$ to describe the transition between different energy levels and Lie algebra operator$I^{\alpha } (\alpha =\pm ,3)$ represent transfers between states at the same level. The details are illustrated in Fig. 1.

Many models have Yangian symmetry, such as Heisenerg models, Hubbard model. In studying the spin chain model, we find that Yangian of a multi-particle system has a good rule if only three particles are considered to interact. We will use spin-$\frac{1}{2}$ XXZ model with periodic boundary condition as an example to explain the rules.

As we know, the Hamiltonian of XXZ spin chain is $H_N= \sum ^N_{i,j=1}h_{ij}=J \sum ^N_{i,j=1} \frac{1}{2}(s_i^+s_j^-+s_i^-s_j^+)+\Delta s^3_is^3_j$. Here we consider the diamagnetic case ($J=1$). When we consider the interaction of XXZ closed spin chain between 1, 2 and N particles (the other three particles interaction law will be presented in the next part), the Lie algebra and Yangian of the system will show a perfect rule and the Hamiltonian of *N* particles system is as follows,

1 $$\begin{aligned} H=h_{12}+h_{N1}-h_{N2}. \end{aligned}$$

When N=3, the eigensystem conditions of the system are: $\lambda _1=\lambda _2=\frac{\Delta }{4},\lambda _3=\lambda _4=\frac{1}{4}(2+\Delta ),\lambda _5=\lambda _6=-\frac{1}{4}(1+\Delta +\sqrt{9+4\Delta ^2-4\Delta }),\lambda _7=\lambda _8=-\frac{1}{4}(1+\Delta -\sqrt{9+4\Delta ^2-4\Delta })$. The corresponding eigenstates of three particles system are as follows,

2 $$ \begin{aligned}{}&|\phi _1\rangle =|\downarrow \downarrow \downarrow \rangle ,\\ &|\phi _2\rangle =|\uparrow \uparrow \uparrow \rangle ,\\ &|\phi _3\rangle =\frac{1}{\sqrt{2}}(|\downarrow \downarrow \uparrow \rangle -|\downarrow \uparrow \downarrow \rangle ) ,\\ &|\phi _4\rangle =\frac{1}{\sqrt{2}}(|\uparrow \downarrow \uparrow \rangle -|\uparrow \uparrow \downarrow \rangle ),\\ &|\phi _5\rangle =\frac{1}{\sqrt{a^2+2}}(a|\uparrow \downarrow \downarrow \rangle +|\downarrow \uparrow \downarrow \rangle +|\downarrow \downarrow \uparrow \rangle ),\\ &|\phi _6\rangle =\frac{1}{\sqrt{2b^2+1}}(b|\uparrow \uparrow \downarrow \rangle +b|\uparrow \downarrow \uparrow \rangle +|\downarrow \uparrow \uparrow \rangle ),\\ &|\phi _7\rangle =\frac{1}{\sqrt{c^2+2}}(c|\uparrow \downarrow \downarrow \rangle +|\downarrow \uparrow \downarrow \rangle +|\downarrow \downarrow \uparrow \rangle ),\\ &|\phi _8\rangle =\frac{1}{\sqrt{2d^2+1}}(d|\uparrow \uparrow \downarrow \rangle +d|\uparrow \downarrow \uparrow \rangle +|\downarrow \uparrow \uparrow \rangle ). \end{aligned} $$

As the number of particles studied grows, The eigenvalues rule of N-particle system are

3 $$\begin{aligned}{}&\lambda _1=\lambda _2=\cdots \cdots =\lambda _{2^{N-2}}=\frac{\Delta }{4},\\ &\lambda _{2^{N-2}+1}=\lambda _{2^{N-2}+2}=\cdots \cdots =\lambda _{2^{N-1}}=\frac{1}{4}(2+\Delta ),\\ &\lambda _{2^{N-1}+1}=\lambda _{2^{N-1}+2}=\cdots \cdots =\lambda _{3*2^{N-2}}=-\frac{1}{4}(1+\Delta +\sqrt{9+4\Delta ^2-4\Delta }),\\ &\lambda _{3*2^{N-2}+1}=\lambda _{3*2^{N-2}+2}=\cdots \cdots =\lambda _{2^N}=-\frac{1}{4}(1+\Delta -\sqrt{9+4\Delta ^2-4\Delta }). \end{aligned} $$

The degeneracies of the four eigenvalues are same ($2^{N-2}$). The eigenvalues of N-particle system have good regularity, and the eigenstates law of N-particle closed XXZ spin chain system can be obtained from the eigenstates of three-particle system. According to the eigenstates of three particles system, we can get the eigenstates of four particles system firstly, the details are as follows,

4 $$\begin{aligned} |\phi _1\rangle \Rightarrow&{\left\{ \begin{array}{ll} |\psi _1\rangle =|\downarrow \downarrow \downarrow \downarrow \rangle \\ |\psi _2\rangle =|\downarrow \downarrow \uparrow \downarrow \rangle \end{array}\right. }\\ |\phi _2\rangle \Rightarrow&{\left\{ \begin{array}{ll} |\psi _3\rangle =|\uparrow \uparrow \downarrow \uparrow \rangle \\ |\psi _4\rangle =|\uparrow \uparrow \uparrow \uparrow \rangle \end{array}\right. }\\ |\phi _3\rangle \Rightarrow&{\left\{ \begin{array}{ll} |\psi _5\rangle =\frac{1}{\sqrt{2}}(|\downarrow \downarrow \downarrow \uparrow \rangle -|\downarrow \uparrow \downarrow \downarrow \rangle )\\ |\psi _6\rangle =\frac{1}{\sqrt{2}}(|\downarrow \downarrow \uparrow \uparrow \rangle -|\downarrow \uparrow \uparrow \downarrow \rangle ) \end{array}\right. }\\ |\phi _4\rangle \Rightarrow&{\left\{ \begin{array}{ll} |\psi _7\rangle =\frac{1}{\sqrt{2}}(|\uparrow \downarrow \downarrow \uparrow \rangle -|\uparrow \uparrow \downarrow \downarrow \rangle )\\ |\psi _8\rangle =\frac{1}{\sqrt{2}}(|\uparrow \downarrow \uparrow \uparrow \rangle -|\uparrow \uparrow \uparrow \downarrow \rangle ) \end{array}\right. }\\ |\phi _5\rangle \Rightarrow&{\left\{ \begin{array}{ll} |\psi _9\rangle =\frac{1}{\sqrt{a^2+2}}(a|\uparrow \downarrow \downarrow \downarrow \rangle +|\downarrow \uparrow \downarrow \downarrow \rangle +|\downarrow \downarrow \downarrow \uparrow \rangle )\\ |\psi _{10}\rangle =\frac{1}{\sqrt{a^2+2}}(a|\uparrow \downarrow \uparrow \downarrow \rangle +|\downarrow \uparrow \uparrow \downarrow \rangle +|\downarrow \downarrow \uparrow \uparrow \rangle ) \end{array}\right. }\\ |\phi _6\rangle \Rightarrow&{\left\{ \begin{array}{ll} |\psi _{11}\rangle =\frac{1}{\sqrt{2b^2+1}}(b|\uparrow \uparrow \downarrow \downarrow \rangle +b|\uparrow \downarrow \downarrow \uparrow \rangle +|\downarrow \uparrow \downarrow \uparrow \rangle )\\ |\psi _{12}\rangle =\frac{1}{\sqrt{2b^2+1}}(b|\uparrow \uparrow \uparrow \downarrow \rangle +b|\uparrow \downarrow \uparrow \uparrow \rangle +|\downarrow \uparrow \uparrow \uparrow \rangle ) \end{array}\right. }\\ |\phi _7\rangle \Rightarrow&{\left\{ \begin{array}{ll} |\psi _{13}\rangle =\frac{1}{\sqrt{c^2+2}}(c|\uparrow \downarrow \downarrow \downarrow \rangle +|\downarrow \uparrow \downarrow \downarrow \rangle +|\downarrow \downarrow \downarrow \uparrow \rangle )\\ |\psi _{14}\rangle =\frac{1}{\sqrt{c^2+2}}(c|\uparrow \downarrow \uparrow \downarrow \rangle +|\downarrow \uparrow \uparrow \downarrow \rangle +|\downarrow \downarrow \uparrow \uparrow \rangle ) \end{array}\right. }\\ |\phi _8\rangle \Rightarrow&{\left\{ \begin{array}{ll} |\psi _{15}\rangle =\frac{1}{\sqrt{2d^2+1}}(d|\uparrow \uparrow \downarrow \downarrow \rangle +d|\uparrow \downarrow \downarrow \uparrow \rangle +|\downarrow \uparrow \downarrow \uparrow \rangle )\\ |\psi _{16}\rangle =\frac{1}{\sqrt{2d^2+1}}(d|\uparrow \uparrow \uparrow \downarrow \rangle +d|\uparrow \downarrow \uparrow \uparrow \rangle +|\downarrow \uparrow \uparrow \uparrow \rangle ) \end{array}\right. } \end{aligned} $$

where $$ a=\frac{1}{2}(1-2\Delta -\sqrt{9+4(\Delta -1)\Delta }), b=\frac{1}{4}(-1+2\Delta -\sqrt{9+4(\Delta -1)\Delta }), c=\frac{1}{2}(1-2\Delta +\sqrt{9+4(\Delta -1)\Delta }), d=\frac{1}{4}(-1+2\Delta +\sqrt{9+4(\Delta -1)\Delta }).$$

During the study, we found that the eigensystem of the model of the first, the second and the last of four particles system can be obtained from the eigensystem of three particles system. From the above research content, we can discover the law of structure that the eigenstates of the four-particle system can be obtained by adding $\uparrow , \downarrow$ at the penultimate position of three-particle engenstates. Five-particle system’s eigenstates are also obtained from four-particle system by adding $\uparrow , \downarrow$ at penultimate position. The same holds true when we extend the scope of the study to all multi-particle system. In the course of studying this system, we can know that the system’s energy levels have a high degree of degeneracy ($2^{N-2}$) and clear regularity.

In this system, the research object is the first, second and the last particle interaction of N-particle. From the previous introduction, we can see that there are two important applications of Yangian: describing symmetry and constructing transition operators. The relations between Lie algebra operators and Yangian operators are as follows:

5 $$\begin{aligned}{}&[I^+,I^-]=2I^3,\quad [I^3,I^+]=I^+,\quad [I^3,I^-]=-I^-,\\ &[I^3,J^+]=J^+,\quad [I^3,J^-]=-J^-,\quad [J^3,I^-]=-J^-,\\ &[J^3,I^-]=-J^-,\quad [I^+,J^-]=2J^3,\quad [I^-,J^+]=-2J^3,\\ &[I^3,J^3]=0,\quad [I^+,J^+]=0,\quad [I^-,J^-]=0,\\ &[J^3,[J^+,J^-]]=0,\quad [J^+,[J^3,J^+]]=0,\quad [J^-,[J^3,J^-]]=0,\\ &[J^+,[J^+,J^-]]+2[J^3,[J^3,J^+]]=0,\quad [J^-,[J^-,J^+]]+2[J^3,[J^3,J^-]]=0. \end{aligned} $$

According to the system’s eigensystem and particles interaction laws of this system, the Lie algebra $I_\alpha ,(\alpha =\pm ,3)$ and Yangian $J_\alpha ,(\alpha =\pm ,3)$ transition relations among eigenstates have the following forms,

6 $$\begin{aligned} I_+=&\sum _{m=0}^{3} \sum _{i=1}^{2^{n-3}-1}[\sqrt{(2^{n-3})^2}|\Psi _{2^{n-3}+m*2^{n-2}}\rangle \langle \Psi _{2^{n-3}+1+m*2^{n-2}}|\\ &+\sqrt{(2^{n-3})^2-(2i-1)}(|\Psi _{2^{n-3}+m*2^{n-2}-i}\rangle \langle \Psi _{2^{n-3}+1+m*2^{n-2}-i}|+|\Psi _{2^{n-3}+m*2^{n-2}+i}\rangle \langle \Psi _{2^{n-3}+1+m*2^{n-2}+i}|)] \\ I_-=&\sum _{m=0}^{3} \sum _{i=1}^{2^{n-3}-1}[\sqrt{(2^{n-3})^2}|\Psi _{2^{n-3}+1+m*2^{n-2}}\rangle \langle \Psi _{2^{n-3}+m*2^{n-2}}|\\ &+\sqrt{(2^{n-3})^2-(2i-1)}(|\Psi _{2^{n-3}+1+m*2^{n-2}-i}\rangle \langle \Psi _{2^{n-3}+m*2^{n-2}-i}|+|\Psi _{2^{n-3}+1+m*2^{n-2}+i}\rangle \langle \Psi _{2^{n-3}+m*2^{n-2}+i}|)] \\ I_3=&\sum _{m=0}^{3} \sum _{i=1}^{2^{n-3}-1}\frac{2i+1}{2}(|\Psi _{2^{n-3}+m*2^{n-2}-i}\rangle \langle \Psi _{2^{n-3}+m*2^{n-2}-i}|-|\Psi _{2^{n-3}+1+m*2^{n-2}+i}\rangle \langle \Psi _{2^{n-3}+1+m*2^{n-2}+i})\\ J_+=&\sum _{m=0}^{3} \sum _{i=1}^{2^{n-3}-1}[\sqrt{(2^{n-3})^2}(|\Psi _{2^n-2^{n-3}-m*2^{n-2}}\rangle \langle \Psi _{2^n-(m+1)*2^{n-2}+1}|+|\Psi _{2^{n-3}+m*2^{n-2}}\rangle \langle \Psi _{(m+1)*2^{n-2}+1}|)\\ &+\sqrt{(2^{n-3})^2+(2i-1)}(|\Psi _{2^n-2^{n-3}-m*2^{n-2}-i}\rangle \langle \Psi _{2^n-(m+1)*2^{n-2}+1-i}|+|\Psi _{2^n-2^{n-3}-m*2^{n-2}+i}\rangle \langle \Psi _{2^n-(m+1)*2^{n-2}+1+i}|)\\ &+\sqrt{(2^{n-3})^2+(2i-1)}(|\Psi _{2^{n-3}+m*2^{n-2}-i}\rangle \langle \Psi _{(m+1)*2^{n-2}+1-i}|+|\Psi _{2^{n-3}+m*2^{n-2}+i}\rangle \langle \Psi _{(m+1)*2^{n-2}+1+i}|)]\\ J_-=&\sum _{m=0}^{3} \sum _{i=1}^{2^{n-3}-1}[\sqrt{(2^{n-3})^2}(|\Psi _{2^n-(m+1)*2^{n-2}+1}\rangle \langle \Psi _{2^n-2^{n-3}-m*2^{n-2}}|+|\Psi _{(m+1)*2^{n-2}+1}\rangle \langle \Psi _{2^{n-3}+m*2^{n-2}}|)\\ &+\sqrt{(2^{n-3})^2+(2i-1)}(|\Psi _{2^n-(m+1)*2^{n-2}+1-i}\rangle \langle \Psi _{2^n-2^{n-3}-m*2^{n-2}-i}|+|\Psi _{2^n-(m+1)*2^{n-2}+1+i}\rangle \langle \Psi _{2^n-2^{n-3}-m*2^{n-2}+i}|)\\ &+\sqrt{(2^{n-3})^2+(2i-1)}(|\Psi _{(m+1)*2^{n-2}+1-i}\rangle \langle \Psi _{2^{n-3}+m*2^{n-2}-i}|+|\Psi _{(m+1)*2^{n-2}+1+i}\rangle \langle \Psi _{2^{n-3}+m*2^{n-2}+i}|)]\\ J_3=&\sum _{m=0}^{3} \sum _{i=1}^{2^{n-3}-1}\frac{2i+1}{2}[(|\Psi _{2^{n-3}+m*2^{n-2}-i}\rangle \langle \Psi _{2^{n-3}+(1+m)*2^{n-2}-i}|-|\Psi _{2^{n-3}+1+m*2^{n-2}+i}\rangle \langle \Psi _{2^{n-3}+(1+m)*2^{n-2}+1+i}|)\\ &+(|\Psi _{2^{n-3}+(1+m)*2^{n-2}-i}\rangle \langle \Psi _{2^{n-3}+m*2^{n-2}-i}|-|\Psi _{2^{n-3}+(1+m)*2^{n-2}+1+i}\rangle \langle \Psi _{2^{n-3}+1+m*2^{n-2}+i}|)] \end{aligned} $$

The above formulas are the concrete representations of Lie algebra and Yangian transition operators, where $|\Psi \rangle$ represents the eigenstate in the system of N particles, and *n* is the number of particles in the system. The Yangian operators express the transition between different states at different energy levels and Lie algebra operators represents the transfer between different states in the same energy level. We will illustrate specific transitions between states in Fig. 1.

Figure 1 shows the energy levels of three-particle interaction (the first, second and the last particles) of *N*-particle closed XXZ spin chain model, it clearly shows the four energy levels of this system and corresponding energy level transitions. The Hamiltonian of this system is $$ H_N=h_{12}+h_{N1}-h_{N2},$$ where $$ h_{ij}= \frac{1}{2}(s_i^+s_j^-+s_i^-s_j^+)+\Delta s^3_is^3_j$$. It is found that the same Hamiltonian can be constructed by another way of XXZ model’s Hamiltonian. As we know, the Hamiltonian of Heisenberg XXZ closed chain model is $$H_{Nxxz}=\sum ^n_{i,j=1}\frac{1}{2}(s_i^+s_j^-+s_i^-s_j^+)+\Delta s^3_is^3_j$$, we can reconstruct the same Hamiltonian in the following way, $$H_{Nxxz}-H_{(N-1)xxz}$$. Different particle number models correspond to different action spaces, so we add the identity matrix. This ensures that the action space is the same and does not destroy the structure of Hamiltonian. Take four particles system for example: $$ H_4=H_{4xxz}-H_{3xxz}=H_{4xxz}-I\otimes H_{3xxz}=h_{12}+h_{41}-h_{42}$$. In the example above, we can get the same Hamiltonian when we put the identity matrix in front of the three-particle Hamiltonian, but if we put the identity matrix behind the three-particle Hamiltonian, we will construct a new system with different laws. Since the identity matrix constructs different system at different locations and correspond to different laws, the research content of this part will be presented in the next section.

## Yangian and eigensystem laws of closed XXZ spin chain of N particles

We have already known the partial particles interaction laws of closed XXZ spin chain model in the last section, in this part we will give all the rules of partial particles interaction in closed XXZ spin chain model. From the form of the Hamiltonian in the previous section, we know that the first, second and the last particles’ interaction have good laws, but the Hamiltonian constructed by the interaction of the first, last and penultimate particles also has similar laws. The Hamiltonian has the following form, $H_N=h_{N1}+h_{N-1,N}-h_{N-1,1}$, the corresponding eigen levels have the same values as Eq. (2) and eigenstates are as following,

7 $$\begin{aligned} |\phi _1\rangle =&|\downarrow \downarrow \downarrow \rangle \Rightarrow {\left\{ \begin{array}{ll} |\psi _1\rangle =|\downarrow \downarrow \downarrow \downarrow \rangle \\ |\psi _2\rangle =|\downarrow \uparrow \downarrow \downarrow \rangle \end{array}\right. } \\ |\phi _2\rangle =&|\uparrow \uparrow \uparrow \rangle \Rightarrow {\left\{ \begin{array}{ll} |\psi _3\rangle =|\uparrow \uparrow \uparrow \uparrow \rangle \\ |\psi _4\rangle =|\uparrow \downarrow \uparrow \uparrow \rangle \end{array}\right. } \\ |\phi _3\rangle =&\frac{1}{\sqrt{2}}(-|\uparrow \downarrow \downarrow \rangle +|\downarrow \uparrow \downarrow \rangle )\Rightarrow {\left\{ \begin{array}{ll} |\psi _5\rangle =\frac{1}{\sqrt{2}}(-|\uparrow \uparrow \downarrow \downarrow \rangle +|\downarrow \uparrow \uparrow \downarrow \rangle )\\ |\psi _6\rangle =\frac{1}{\sqrt{2}}(-|\uparrow \downarrow \downarrow \downarrow \rangle +|\downarrow \downarrow \uparrow \downarrow \rangle ) \end{array}\right. }\\ |\phi _4\rangle =&\frac{1}{\sqrt{2}}(-|\uparrow \downarrow \uparrow \rangle +|\downarrow \uparrow \uparrow \rangle )\Rightarrow {\left\{ \begin{array}{ll} |\psi _7\rangle =\frac{1}{\sqrt{2}}(-|\uparrow \uparrow \downarrow \uparrow \rangle +|\downarrow \uparrow \uparrow \uparrow \rangle )\\ |\psi _8\rangle =\frac{1}{\sqrt{2}}(-|\uparrow \downarrow \downarrow \uparrow \rangle +|\downarrow \downarrow \uparrow \uparrow \rangle ) \end{array}\right. }\\ |\phi _5\rangle =&\frac{1}{\sqrt{a^2+2}}(a|\uparrow \uparrow \downarrow \rangle +|\uparrow \downarrow \uparrow \rangle +|\downarrow \uparrow \uparrow \rangle )\Rightarrow {\left\{ \begin{array}{ll} |\psi _9\rangle =\frac{1}{\sqrt{a^2+2}}(a|\uparrow \uparrow \uparrow \downarrow \rangle +|\uparrow \uparrow \downarrow \uparrow \rangle +|\downarrow \uparrow \uparrow \uparrow \rangle )\\ |\psi _{10}\rangle =\frac{1}{\sqrt{a^2+2}}(a|\uparrow \downarrow \uparrow \downarrow \rangle +|\uparrow \downarrow \downarrow \uparrow \rangle +|\downarrow \downarrow \uparrow \uparrow \rangle ) \end{array}\right. } \\ |\phi _6\rangle =&\frac{1}{\sqrt{1+2b^2}}(b|\uparrow \downarrow \downarrow \rangle +b|\downarrow \uparrow \downarrow \rangle +|\downarrow \downarrow \uparrow \rangle )\Rightarrow {\left\{ \begin{array}{ll} |\psi _{11}\rangle =\frac{1}{\sqrt{1+2b^2}}(b|\uparrow \uparrow \downarrow \downarrow \rangle +b|\downarrow \uparrow \uparrow \downarrow \rangle +|\downarrow \uparrow \downarrow \uparrow \rangle )\\ |\psi _{12}\rangle =\frac{1}{\sqrt{1+2b^2}}(b|\uparrow \downarrow \downarrow \downarrow \rangle +b|\downarrow \downarrow \uparrow \downarrow \rangle +|\downarrow \downarrow \downarrow \uparrow \rangle ) \end{array}\right. } \\ |\phi _7\rangle =&\frac{1}{\sqrt{c^2+2}}(c|\uparrow \uparrow \downarrow \rangle +|\uparrow \downarrow \uparrow \rangle +|\downarrow \uparrow \uparrow \rangle )\Rightarrow {\left\{ \begin{array}{ll} |\psi _{13}\rangle =\frac{1}{\sqrt{c^2+2}}(c|\uparrow \uparrow \uparrow \downarrow \rangle +|\uparrow \uparrow \downarrow \uparrow \rangle +|\downarrow \uparrow \uparrow \uparrow \rangle )\\ |\psi _{14}\rangle =\frac{1}{\sqrt{c^2+2}}(c|\uparrow \downarrow \uparrow \downarrow \rangle +|\uparrow \downarrow \downarrow \uparrow \rangle +|\downarrow \downarrow \uparrow \uparrow \rangle ) \end{array}\right. }\\ |\phi _8\rangle =&\frac{1}{\sqrt{2d^2+1}}(d|\uparrow \downarrow \downarrow \rangle +d|\downarrow \uparrow \downarrow \rangle +|\downarrow \downarrow \uparrow \rangle )\Rightarrow {\left\{ \begin{array}{ll} |\psi _{15}\rangle =\frac{1}{\sqrt{2d^2+1}}(d|\uparrow \uparrow \downarrow \downarrow \rangle +d|\downarrow \uparrow \uparrow \downarrow \rangle +|\downarrow \uparrow \downarrow \uparrow \rangle )\\ |\psi _{16}\rangle =\frac{1}{\sqrt{2d^2+1}}(d|\uparrow \downarrow \downarrow \downarrow \rangle +d|\downarrow \downarrow \uparrow \downarrow \rangle +|\downarrow \downarrow \downarrow \uparrow \rangle ) \end{array}\right. } \end{aligned} $$

where $a=\frac{1}{2}(1-2\Delta -\sqrt{9+4(\Delta -1)\Delta }), b=\frac{1}{4}(-1+2\Delta -\sqrt{9+4(\Delta -1)\Delta }), c=\frac{1}{2}(1-2\Delta +\sqrt{9+4(\Delta -1)\Delta }), d=\frac{1}{4}(-1+2\Delta +\sqrt{9+4(\Delta -1)\Delta }).$ The eigenvalues of this system are identical to the eigenvalues of “Yangian of closed XXZ spin chain of N particles” section, Eq. (2). Through the above eigenstates Eq. (7), we can find a very similar place with previous formulas of Eqs. (3) and (4). They have the same coefficients and similar structures, but on closer inspection, the eigenstates are different. Similarly, we can generalize the multi-particle eigensystem from the first three-particle eigensystem (Eq. 7), when we get four particles eigenstates from three-particle eigenstates, we need to put operators $\uparrow , \downarrow$ in the second positive, same thing with five particles. By contrast, we can still derive the Hamiltonian for this system using the Heisenberg closed XXZ spin chain model: $H_N=H_{Nxxz}-H_{(N-1)xxz}\otimes I$.

Through the analysis of the Hamiltonian of the system we studied, it is found that the interaction of three adjacent particles in the multi-particle system has good laws. Not only do we know how the first, the second and the last particles interact, but we also know how the first, the last and the penultimate particle interact. In a multi-particle system, the eigenstates of the interactions of the other three adjacent particles are illustrated by the following Fig. 2.

Figure 2 shows the interaction models of any three adjacent particles in the N-body particles system. The heavy solid line indicates that the two particles’ interaction terms are added ($h_{ij}$), and the dotted line indicates that the two particles’ interaction terms are subtracted ($-h_{ij}$). In Fig. 2, we use double arrows to represent systems with Hamiltonian of $H_N=h_{1,2}+h_{N,1}-h_{N,2}$. This system consists of 1, 2 and *N* particles. By adding operators $\uparrow , \downarrow$ to the penultimate position of the eigenstates of the first three-particle model, the eigenstates ($H_N=h_{1,2}+h_{N,1}-h_{N,2}$) of the multi-particle system can be obtained. The dotted line arrows represent the system of 1, *N* and $N-1$ particles ($H_N=h_{N,1}+h_{N-1,N}-h_{N-1,1}$), for *N* particle systems, we can obtain the corresponding eigenstate by adding operators $\uparrow , \downarrow$ in the second positive and we mark this case with a dotted line. In Fig. 2, there are two other generalizations, one is to add operators $\uparrow , \downarrow$ at the front of the eigenstates, consisted of the $N, N-1$ and $N-2$ particles, it is represented by solid line arrow, and the other is to add an operator at the last position of the eigenstates, represented by dotted arrows, the three adjacent particles studied are 1, 2, 3; 2, 3, 4; 3, 4, 5; ...... particles. All of them have the same eigenvalue case as Eq. (2), the eigenstates are different from each other, but the law is the same.

In “Introduction” section, we study the 1, 2 and *N* particles interaction terms model in closed Heisenberg XXZ spin chain model of *N* particle system, the rules of eigensystem of this model are studied. The degenerate case of the system is $2^{N-2}$, the corresponding Yangian are studied, it is specifically expressed as Eq. (6) and Fig. 1. In this section, on the basis of “Introduction” section, we examine in detail the eigensystems laws of any three adjacent particle interactions, the composition of the Hamiltonian and its relationship to the closed XXZ spin chain model are also be researched. According to the studied laws, we can get the eigensystem of the model composed of any three adjacent particle system in *N* particles closed XXZ spin chain model system. In the next section, we will study the corresponding laws of other spin chain models in the Heisenberg model and extending the scope of the study to high-spin systems.

## Studies on the laws of XXX model, XXZ model with other conditions and other models

In the previous two parts, we studied the relevant laws of the closed XXZ spin chain model, in this part, we extended the research scope to the open and closed XXX spin chain model, XY spin chain and Ising model. We will also consider the next nearest neighbor particle interaction, the twist boundary conditions and spin-1 system laws of these models.

The periodic boundary conditions which required $S_{N+1}\equiv S_1$ (*N* is particle’s number)are often considered in the study of closed Heisenberg spin chain studies, then the twist boundary condition was introduced. In 1990, Bill Sutherland and B. Sriram Shastry considered a one-dimensional quantum many-body system on a ring of length *L* with *M* particles^29^, each with a charge of q. When the ring is crossed by a magnetic flux of strength, according to the Aharonov-Bohm effect which is discussed by Beyers and Yang on a quantum many-body system^30^, the twist boundary condition $\varPsi _N(x_1,...,x_j+L,...x_N)=e^{i\varPhi }\varPsi _N(x_1,...,x_j,...x_N)$ is generated. Here we will also study the Heisenberg model with twist boundary conditions. When we consider the two ends of the chain are attached with a twist of angle $\emptyset$ around the z-axis, the boundary condition is $s^+_{n+1}=s_1^+ e^{i\emptyset }, s^-_{n+1}=s_1^- e^{-i\emptyset }, s^z_{n+1}=s_1^z.$ Thus the Hamiltonian of XXZ model is $H_{xxzt}=\sum _{i=1}^{N-1}\frac{1}{2}(s_i^+s_{i+1}^-+s_i^-s_{i+1}^+)\Delta s_i^z s_{i+1}^z+\frac{1}{2}(s_N^+s_1^-e^{-i\emptyset }+s_N^-s_1^+e^{i\emptyset }+\Delta s_N^zs_1^z).$ According to this boundary term, $H_{xxzt}$ has neither translational symmetry nor isotropy, the translational invariance can be restored if we twist all neighboring bonds in the chain by an angle $\phi =\frac{\emptyset }{N}$. The Hamiltonian can be rewrite the forms $H_t(\phi )=\sum _{i=1}^{N}\frac{1}{2}(s_i^+s_{i+1}^-e^{-i\phi }+s_i^-s_{i+1}^+e^{i\phi })+\Delta s_i^zs_{i+1}^z$^31^. In “Yangian of closed XXZ spin chain of N particles” section, we considered the periodic boundary, in this section, we will study *N* particles closed XXZ spin chain with twist boundary condition (TBC), the Hamiltonian have the following forms: $H_N-I\otimes H_{N-1}=h_{1,2}+h_{N,1}-h_{N,2}$ or $H_N-H_{N-1}\otimes I=h_{N,1}+h_{N-1,N}-h_{N-1,1}$, it is found that the eigenstates law of the interaction model of three neighboring particles in N particles system extended from three-particle systems are the same as closed XXZ spin chain, except for the specific representation of the eigenstates are different. the degeneracies of the model are $2^{N-2}$, the Yangian has the same forms as Fig. 1.

The open XXZ spin chain system is also considered in this paper. It constructed Hamiltonian in the same way as the “Yangian of closed XXZ spin chain of N particles” section, the Hamiltonian of the model is $H_3=H_{3xxz}-I\otimes H_{2xxz}=h_{12}$ or $H_3=H_{3xxz}-H_{2xxz} \otimes I = h_{N-1,N}$, its corresponding eigenvalues are $\frac{1}{4}(-2-\Delta ), \frac{1}{4}(2-\Delta ), \frac{\Delta }{4}$, the degeneracies are $2^{N-2},2^{N-2},2^{N-1}$. The eigenstates are still extended by adding operators $\uparrow ,\downarrow$ in the second, penultimate, first or last position. Next, we will consider the open XXZ spin chain system with next-nearest neighbor case, the Hamiltonian’s constructions is similar to the open chain case, the Hamiltonian has the forms: $H_3=H_{3xxz}-I\otimes H_{2xxz}=h_{12}+h_{13}$ or $H_3=H_{3xxz}-H_{2xxz} \otimes I = h_{N-1,N}+h_{N-2,N}$, the degeneracies are $2^{N-2},2^{N-2},2^{N-1}$. The model is extended in the same way. The open XXZ spin chain is similar to the open XXZ spin chain with next-nearest neighbor case in the eigensystem and Yangian.

In this paper, we also consider the other models, such as XXX, XY and Ising models. When we study the closed spin chain XXX model, the degeneracies of the system are $2^{N-2},2^{N-2},2^{N-1}$, and the Hamiltonian’s constructions are $H_N-I\otimes H_{N-1}=h_{1,2}+h_{N,1}-h_{N,2}$ or $H_N-H_{N-1}\otimes I=h_{N,1}+h_{N-1,N}-h_{N-1,1}$, the eigenvalues are $-\frac{5}{4},-\frac{3}{4}, \frac{1}{4}$, for the system of $H_N-I\otimes H_{N-1}=h_{1,2}+h_{N,1}-h_{N,2}$, the corresponding eigenstates of the first three-particle system are $\phi _1=\frac{1}{\sqrt{6}}(-2|\uparrow \downarrow \downarrow \rangle +|\downarrow \uparrow \downarrow \rangle +|\downarrow \downarrow \uparrow \rangle ), \phi _2=\sqrt{\frac{2}{3}}(-\frac{1}{2}|\uparrow \uparrow \downarrow \rangle -\frac{1}{2}|\uparrow \downarrow \uparrow \rangle +|\downarrow \uparrow \uparrow \rangle ),\phi _3=\frac{1}{\sqrt{2}}(-|\downarrow \uparrow \downarrow \rangle +|\downarrow \downarrow \uparrow \rangle ), \phi _4=\frac{1}{\sqrt{2}}(-|\uparrow \uparrow \downarrow \rangle +|\uparrow \downarrow \uparrow \rangle ), \phi _5=\frac{1}{\sqrt{3}}(|\uparrow \downarrow \downarrow \rangle +|\downarrow \uparrow \downarrow \rangle +|\downarrow \downarrow \uparrow \rangle ), \phi _6=\frac{1}{\sqrt{3}}(|\uparrow \uparrow \downarrow \rangle +|\uparrow \downarrow \uparrow \rangle +|\downarrow \uparrow \uparrow \rangle ),\phi _7=|\downarrow \downarrow \downarrow \rangle ,\phi _8=|\uparrow \uparrow \uparrow \rangle$, the eigenstates’ generalization to multi-particle system is to add operators $\uparrow or \downarrow$ in the penultimate position. In the second case $H_N-H_{N-1}\otimes I=h_{N,1}+h_{N-1,N}-h_{N-1,1}$, the energy levels are the same as above, but the specific expression of eigenstates are different, which is generalized by adding an operator $\uparrow or \downarrow$ at the second position. The eigensystem laws of extended to N particle system is the same as that of closed XXZ spin chain model. Similarly, closed XXX spin chain with twist boundary condition (TBC) has the same laws as closed XXZ spin chain with TBC, the degeneracies of the system are $2^{N-2},2^{N-2},2^{N-2},2^{N-2}$, the Hamiltonian’s form are $H_N-I\otimes H_{N-1}=h_{1,2}+h_{N,1}-h_{N,2}$ or $H_N-H_{N-1}\otimes I=h_{N,1}+h_{N-1,N}-h_{N-1,1}$. The generalization of eigenstates are the same.

For the open XXX spin chain model, the Hamiltonian is $H=\sum _{i=1}^{N}\frac{1}{2}(s_i^+s_j^-+s_i^-s_j^+)+\Delta s_i^3s_j^3$, when we construct the Hamiltonian in the way described above in “Yangian of closed XXZ spin chain of N particles” section, the rules of this system’s Hamiltonian are $H_N-I\otimes H_{N-1}=h_{12}$ or $H_N-H_{N-1} \otimes I=h_{N-1,N}$, the degeneracies are $2^{N-2}, 3*2^{N-2}$, the eigenvalues of $H_N-I\otimes H_{N-1}=h_{12}$ are $-\frac{3}{4}, \frac{1}{4}$, eigenstates of the first three particles are $\psi _1=\frac{1}{\sqrt{2}}(-|\uparrow \uparrow \uparrow \rangle +|\downarrow \uparrow \downarrow \rangle ), \psi _2=\frac{1}{\sqrt{2}}(-|\uparrow \downarrow \uparrow \rangle +|\downarrow \uparrow \uparrow \rangle ), \psi _3=|\downarrow \downarrow \downarrow \rangle , \psi _4=|\downarrow \downarrow \uparrow \rangle , \psi _5=\frac{1}{\sqrt{2}}(|\uparrow \downarrow \downarrow \rangle +|\downarrow \uparrow \downarrow \rangle ), \psi _6=\frac{1}{\sqrt{2}}(|\uparrow \downarrow \uparrow \rangle +|\downarrow \uparrow \uparrow \rangle ), \psi _7=|\uparrow \uparrow \downarrow \rangle , \psi _8=|\uparrow \uparrow \uparrow \rangle$. The eigenstates of the multiparticle system are add operators $\uparrow or \downarrow$ at the penultimate position just like Eq. (4). And for $H_N-H_{N-1} \otimes I=h_{N-1,N}$ system, they have the same eigenvalues, but the eigenstates are different and when extended to multi-particle system, the operators $\uparrow , \downarrow$ can be added to the positive second position. Its generalizations are the same as open XXZ spin chain model. For the next neighbor case, from the initial three-particle system to the multi-particle system, the law of the eigensystem of the adjacent three particles model is the same as that of the XXZ model, the degeneracies are $2^{N-2},2^{N-2},2^{N-1}$, the Hamiltonian has the same forms as XXZ model: $H_3=H_{3xxx}-I\otimes H_{2xxx}=h_{12}+h_{13}$ or $H_3=H_{3xxx}-H_{2xxx} \otimes I = h_{N-1,N}+h_{N-2,N}$. The generalization mode of these models’ eigensystem is the same as that of XXZ model.

In addition, XY, Ising model and spin-1 system of these systems were also studied. Their eigensystems have the same rules of generalization. In the closed chain with periodic boundary conditions of the above models, the Hamiltonian of any three adjacent particle models is $H_N-I\otimes H_{N-1}=h_{1,2}+h_{N,1}-h_{2,N} or H_N-H_{N-1}\otimes I=h_{N,1}+h_{N-1,N}-h_{N-1,1}$. For an open-chain condition of the above models, the Hamiltonian of any three adjacent particle models are $H_3=H_{3xxx}-I\otimes H_{2xxx}=h_{12}$ or $H_3=H_{3xxx}-H_{2xxx} \otimes I = h_{N-1,N}$. For open chain model containing next nearest neighbor of above models, the Hamiltonian of any three adjacent particle models are $H_3=H_{3xxx}-I\otimes H_{2xxx}=h_{12}+h_{13}$ or $H_3=H_{3xxx}-H_{2xxx} \otimes I = h_{N-1,N}+h_{N-2,N}$. The corresponding eigensystem of each system is not the same, but the promotion methods are same.

## Summary

In this paper, we consider the interaction model of any three adjacent particles of Heisenberg spin chain systems. Firstly, we consider the first, second and the last particles model of the closed XXZ spin chain, the system has four sets of eigenvalues and their degeneracies are $2^{N-2}$. Through the eigensystem of these three particles, we can get the eigensystem of the system composed of the first, second and the last particle in the muli-particle system. The Hamiltonian of the same three adjacent particle systems can be constructed by a closed XXZ spin chain model in the form of $H_N=H_{Nxxz}-I\otimes H_{(N-1)xxz}$, the Yangian rule of the model is shown in Fig. 1. Secondly, we study the eigensystem and Yangian laws of other similar three particles systems of the closed XXZ spin chain model. In a multi-particle system, the constitutive laws of the interaction model between any close three particles and the generalization laws of their eigenstates have also been found. The generalizations of the model are illustrated in detail in Fig. 2. Last, we also studied the open-chain, next-nearest neighbor and twist boundary conditions of XXZ spin chain model. Corresponding research results have also been obtained for the XXZ spin chain system in the above case. The XXX, XY, Ising model and the spin-1 chain system are also be discussed. According to the research, we found that the eigensystems of these systems are generalized to N particle systems in the same way as the XXZ model.

## References

[1] Loebbert F (2016). Lectures on Yangian Symmetry. J. Phys. A Math. Theor..

[2] Molev AI (2003). Yangians and their applications. Handb. Algebra.

[3] Molev A, Nazarov M, Ol’shanskii G (1996). Yangians and classical Lie algebras. Russ. Math. Surv..

[4] Belavin A (1992). A direct calculation of the spectrum of masses in an integrable model from the Hopf-algebra symmetry. Phys. Lett. B.

[5] Drinfeld VG (1985). Hopf algebras and the quantum Yang–Baxter equation. Sov. Math. Dokl..

[6] Drinfeld, V. G. *Proceedings of International Congress Mathematicians* 798–820 (AMS, 1987).

[7] Drinfeld VG (1988). Quantum Groups. J. Sov. Math..

[8] Stukopin V (2019). Drinfeld Yangian of the queer Lie superalgebra $sq_1$. J Phys. Conf. Ser..

[9] Drinfeld V (1988). A new realization of Yangians and quantized affine algebras. Sov. Math. Dokl..

[10] Molev, A. *Yangians and Classical Lie Algebras. Mathematical Surveys and Monographs*, Vol. 143 (American Mathematical Society, 2007).

[11] Kulish, P. P. & Sklyanin, E. K. Quantum spectral transform method: Recent developments. In *Integrable Quantum Field Theories* 61–119 (Springer, 1982).

[12] Takhtajan LA, Faddeev LD (1979). Quantum inverse scattering method and the Heisenberg XYZ-model. Russ. Math. Surv..

[13] Molev, A. I. Gelfand–Tsetlin bases for classical Lie algebras. In: *Handbook of Algebra*, Vol. 4. 109–170 (Elsevier, 2006).

[14] Wendlandt C (2018). The R-matrix presentation for the Yangian of a simple lie algebra. Commun. Math. Phys..

[15] Nazarov M (1991). Quantum Berezinian and the classical Capelli identity. Lett. Math. Phys..

[16] Nazarov, M. Yangians and Capelli identities. In *Kirillov’s Seminar on Representation Theory* (Am. Math. Soc. Transl.), Vol. 181, 139–163 (American Mathematical Society, 1998).

[17] Nazarov M, Tarasov V (1994). Yangians and Gelfand–Zetlin bases. Publ. Res. Inst. Math. Sci..

[18] Belliard S, Grosjean N, Pimenta RA, Avan J, Karaiskos N (2014). Scattering matrices in the sl(3) twisted yangian. J. Stat. Mech. Theory Exp..

[19] Palla L (2011). Yangian symmetry of boundary scattering in ads/cft and the explicit form of bound state reflection matrices. J. High Energy Phys..

[20] Frassek R (2020). Oscillator realisations associated to the d-type yangian: Towards the operatorial q-system of orthogonal spin chains. Nucl. Phys. B.

[21] Molev A (2013). Feigin–Frenkel center in types B, C and D. Invent. Math..

[22] Molev, A. I., Mukhin, E. E. Yangian characters and classical W-algebras. In *Conformal Field theory, Automorphic Forms and Related Topics*. *Contributions in Mathematical and Computational Sciences*, Vol. 8, pp. 287–334 (Springer, 2014).

[23] Molev AI, Mukhin EE (2017). Eigenvalues of Bethe vectors in the Gaudin model. Theor. Math. Phys..

[24] Gross DJ, Neveu A (1974). Dynamical symmetry breaking in asymptotically free field theories. Phys. Rev. D..

[25] Murakami S, Göhmann F (1997). Yangian symmetry and quantum inverse scattering method for the one-dimensional Hubbard model. Phys. Lett. A.

[26] Uglov DB, Korepin VE (1994). The Yangian symmetry of the Hubbard model. Phys. Lett. A.

[27] Göhmann F, Inozemtsev V (1996). The Yangian symmetry of the Hubbard models with variable range hopping. Phys. Lett. A.

[28] Haldane FDM, Ha ZNC, Talstra JC, Bernard D, Pasquier V (1993). Yangian symmetry of integrable quantum chains with long-range interactions and a new description of states in conformal field theory. Phys. Rev. Lett..

[29] Sutherland B, Shastry BS (1990). Adiabatic transport properties of an exactly soluble one-dimensional quantum many-body problem. Phys. Rev. Lett..

[30] Beyers N, Yang CN (1961). Theoretical considerations concerning quantized magnetic flux in superconducting cylinders. Phys. Rev. Lett..

[31] Fath G, Solyom J (1993). Isotropic spin-1 chain with twisted boundary condition. Phys. Rev. B.

